# Fabrication and mechanical/biological evaluations of dissolving bird-bill microneedle arrays

**DOI:** 10.1007/s13346-024-01757-w

**Published:** 2024-12-09

**Authors:** Natsumi Amano, Yuusei Takaki, Harunori Takei, Masaaki Matsuo, Masaya Hara, Yasunori Tashiro, Takahiro Oniki, Takahiro Ito, Tomohiro Hikima

**Affiliations:** 1 Department of Bioscience and Bioinformatics, Kyushu Institute of Technology, 680-4 Kawazu, Iizuka, Fukuoka 820-8502 Japan; 2 Department of Systems Design and Informatics, Kyushu Institute of Technology, 680-4 Kawazu, Iizuka, Fukuoka 820-8502 Japan; 3 Mishima Kosan Co., Ltd., 2-1-15 Edamitsu, Yahatahigashi, Kitakyushu, 805-0002 Japan

**Keywords:** Bird-bill microneedle, Dissolving microneedle array, Transdermal drug delivery, Biocompatible polymer, Polyvinylpyrrolidone

## Abstract

Coated microneedles (MNs) have several disadvantages, including limited drug doses, decreased skin puncture ability due to drug coating, and a risk of clogging and infection due to repeated application. We aimed to fabricate a dissolving bird-bill MN (dBB MN) with a vertical groove between two thin plate-shaped needles and needle pedestal. Moreover, we evaluated its ability to transdermally deliver a large-molecular-weight insulin into the systemic circulation. Hydrogels with various concentrations of polyvinylpyrrolidone (PVP) or sodium hyaluronate (HA) were prepared, and dBB MN arrays were fabricated by micromolding under negative pressure for potential mass production. The needle height of the dBB MN was at the maximum when the hydrogel was 25 w/w% PVP, with a viscosity of 8–9 Pa∙s. Furthermore, the buckling force of dBB MNs made from 25 w/w% PVP was 130.6 ± 51.0 mN, which increased to 195.6 ± 65.3 mN when insulin was added at 1 w/w%. The skin insertion ability of dBB MN was investigated using swain skin, with micro-holes were confirmed on the skin surface. dBB MN showed biphasic dissolution in the skin; the plate-shaped needles were immediately dissolved within 10 min, while the needle pedestal was slowly dissolved over 180 min. The blood glucose concentration in diabetic rats decreased slowly and significantly after a 3-h application of the insulin-loaded dBB MN array. Therefore, the dBB MN array demonstrated sufficient ability to puncture skin and transdermally deliver a large-molecular-weight drug into the systemic circulation. These findings suggest that the dBB MN array holds promise as a minimal invasive drug delivery platform, with potential applications in improving patient adherence and expanding access to essential therapies, particularly in resource-limited settings.

**Graphical abstract:**

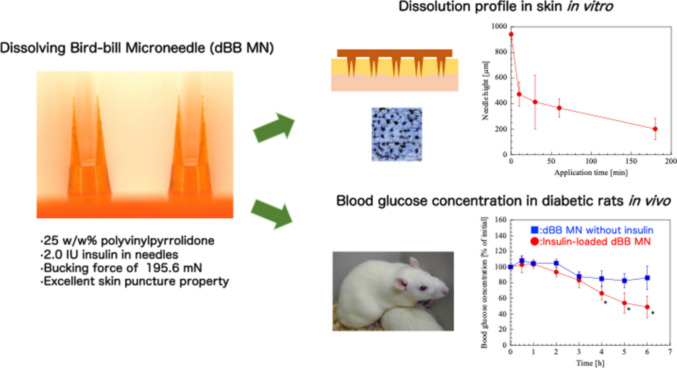

## Introduction

Microneedle (MN) arrays are a physical penetration drug-delivery enhancement method, allowing delivery through the stratum corneum (SC) into the body. The MN array comprises hundreds of micrometer-high MNs, and has the advantage of being a painless device that can be self-administrated by patients. MNs can be classified into five types: hollow, solid, coated, dissolving, and hydrogel-forming MNs. They can be also used for diagnosis and therapeutic drug monitoring [1–3].

Dissolving MNs (dMNs) are fabricated from various biodegradable and biocompatible macro- and high-molecular-weight polymers such as sodium chondroitin sulfate [4], sodium hyaluronate (HA) [5], and poly-γ-glutamic acid [6]. Synthetic biocompatible polymers, such as polyvinyl alcohol (PVA) and polyvinyl pyrrolidone (PVP), also commonly used as materials to construct dMNs [7–9]. dMNs are expected to quickly dissolve in the skin after skin insertion and enable efficient and complete penetration of drugs encapsulated in the MNs across the SC. In addition, they are single-use and incinerable devices. dMNs can contribute to the spread of vaccination in areas with insufficient physicians [10]. However, the release profile of drugs from dMNs and needle hardness depends on the dissolving and degradable properties of the polymers in the skin. The needle hardness of dMNs can be improved by adding additives such as nonionic surfactants [11]; however, it generally tends to be lower than that of other types of MNs. An additional disadvantage of dMN arrays is the limited amount of drug which can be delivered. In the case of hollow and solid MN arrays, drug dosage can be increased without changing the shape of the needle. The dMN array can be modified to influence the structural factors, including the number of needles and needle density, to increase drug dosage.

dMNs generally have conical and pyramidical shapes, which ensure the ability of skin insertion and facilitate the manufacturing process of casting/molding to extract the dMN from the female mold. Researchers have developed various manufacturing processes to fabricate dMNs with pointed tips without a mold. Lithography was performed to prepare maltose dMNs [12]. The prepared MNs had a high aspect ratio and different shapes but were limited to polymers and drugs that do not denature at high temperatures. Kim et al. previously reported a method called droplet-born air blowing, which controlled the amount of drug delivered by the pressure and time of the droplet dispenser and formed an MN shape by blowing air [13]. Inkjet printing [14], 3D printing [15], and spray-filled molding [16] have also been reported; however, most researchers have used microcasting/micromolding. To manufacture dMNs by molding, the polymer hydrogel should be of low viscosity enough to be poured into a female mold that replicates the MNs shape, and the tips of the dMNs should be fine and hard enough to puncture the skin.

Bird-bill MNs reportedly overcome the disadvantages of coated MNs [17]. Bird-bill MNs have a vertical groove between two thin plate-shaped needles; consequently, skin insertion ability is improved, and the amount of drug that is not only coated on the needle, but also filled in the vertical groove, is increased. However, it is difficult to fabricate the dissolving bird-bill (dBB) MNs to ensure geometric complexity. Therefore, we aimed to fabricate a dBB MN array by microcasting/micromolding with mass-production capability, improved skin puncture properties, and sufficient hardness for insertion into the skin.

## Materials and methods

### Materials

Polyvinylpyrrolidone K90 (PVP, average molecular weight; 360,000, special grade) and sodium hyaluronate FCH-80 (HA, molecular weight; 600,000–1,000,000, cosmetic grade) to fabricate dBB MN arrays were purchased from Fujifilm Wako Pure Chemical Co. (Osaka, Japan) and Kikkoman Biochemifa Co. (Tokyo, Japan), respectively. Bovine pancreatic insulin and sodium fluorescein (FL) as model drugs were purchased from Sigma-Aldrich Co. (Tokyo, Japan). Streptozotocin (STZ) and other reagents were purchased from Fujifilm Wako Pure Chemical Co.

### Manufacturing of a dBB MN array

**Fig. 1:**
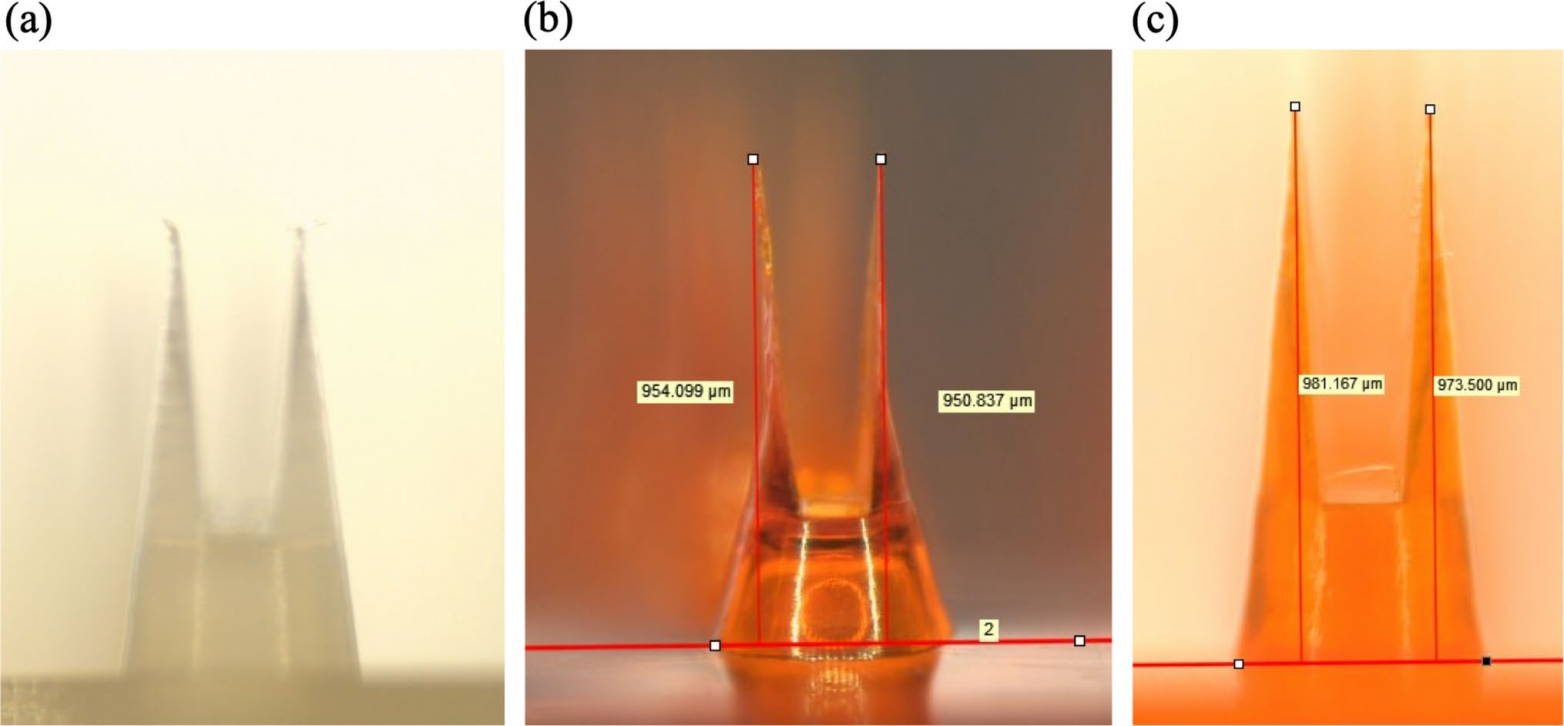
Photographs of (**a**) bird-bill microneedle (MN) original male mold, (**b**) 4% HA dBB MN, and (**c**) 25% PVP dBB MN. The original male mold was fabricated using injection molding, as previously described [17]. dBB MNs contained 0.5 w/w% fluorescein as the model drug

A female mold from polydimethylsiloxane (PDMS) was prepared for the dBB MN array designed to have 52 needles / the base of 0.44 cm^2^ with 1000 µm needle height (700 µm groove and 300 µm needle-pedestal) (original male mold, Fig. 1a). After submerging the original mold into a container filled with PDMS resin and hardener (Sylgard^®^ 184, Dow Chemical Co., MI, USA), the container was put under reduced pressure for 5 min using a vacuum pump Da-20D (Ulvac Kiko Inc., Miyazaki, Japan) to release the air from the PDMS, and subsequently kept at 80 ˚C for 1 h.

HA and PVP hydrogels were prepared at concentrations of 1–4 w/w% and 15–30 w/w%, respectively. The viscosities of the hydrogels were measured using a tuning-fork vibration viscometer SV-10 (viscosity measurement range; 0.3–10,000 mPa∙s, A&D Co., Tokyo, Japan). The hydrogel (0.8–1.0 g) was placed into the PDMS mold and vacuumed at 9.5 torr for 20 min using Da-20D. The air remaining at the tip of the needle after filling the hydrogel was not removed from inside the female mold, even after centrifugation [18]. Therefore, in this study, a vacuum process was used to fill the hydrogel in the mold. The dBB MN array was dried in a desiccator OH-3S (As One Co., Osaka, Japan) at a temperature of 25˚C and humidity of 20% for 24–48 h. The dBB MN arrays were observed under a Leica MC170 HD digital microscope (Leica Microsystems GmbH, Wetzlar, Germany). After measuring the needle height and dBB MN array weight, the array was stored in a desiccator. To determine the amount of insulin contained in the dBB MNs, the weight of all needles on the base of MN array was measured, and confirmed to be 2.08 ± 0.09 mg. The quantity of insulin from bovine pancreas required to lower blood glucose concentrations (BGCs) in diabetic rats (SD strain) is 0.2–5 IU per dMN [19, 20]. Thus, the insulin concentration in the hydrogel was set at 1.0 ww% so that the needles contained at least 0.080 mg (2.0 IU, equaling 25.7 IU/mg in bovine). The other model drug FL was added to be the concentration of 0.5 w/w%, as it could be detected in the following experiments in vitro. The insulin-loaded hydrogels were prepared under ice-cold conditions because heat was generated by mixing PVP and distilled water (DW) and the insulin-loaded dBB MN arrays were prepared immediately before the experiments to prevent insulin denaturation.

### Dissolution experiments of dBB MN

FL-loaded dBB MN (4% HA and 25% PVP) was placed in 50 mL phosphate-buffered saline (PBS, Sigma-Aldrich Co.) preheated at 37 °C. PBS solutions of 500 µL were collected at 1 min intervals until the MN array was completely dissolved. FL concentrations were assayed using a fluorescein spectrometer FP-8550 (excitation wavelength; 495 nm, fluorescent wavelength; 520 nm, temperature; 25 ˚C, Jasco, Tokyo, Japan). We subsequently confirmed that the fluorescent intensity of FL was not affected by the presence or absence of PVP in the PBS.

### Evaluation of insertion capability of dBB MN

The buckling forces of three types of dBB MNs, 4% HA, 25% PVP, and 25% PVP + 1% insulin, were measured using a compression testing machine MCT-510 (Shimadzu Co., Kyoto, Japan) with a loading speed of 20.7 mN/s and a maximum compression force of 1000 mN. The compression test was repeated thrice using a single needle and different needles.

The insertion characteristics of the dMN were evaluated using an artificial skin model, Parafilm^®^ [21]. After dBB MN was applied to the eight layers of the Parafilm^®^ M (American National Can Co., IL, USA) with the total thickness of 1200 µm using the thumb or the spring-driven applicator (5.3 m/s, 17–19 N) for 10 s, the number of micro-holes created in each layer was counted.

Swine skin (landrace strain) with a 2–3 mm thickness as a biological skin model was purchased from DARD Co. (Tokyo, Japan). The skin was placed on a Kimwipe^®^ (Nippon Paper Crecia, Co., Tokyo, Japan) and moistened with DW throughout the experiment. FL-loaded dBB MN (25% PVP) array was punctured into the skin with the thumb for 10 s. After 10, 30, 60, and 180 min, the skin surface was photographed, and the height of the needles was measured using a digital microscope.

### In vivo experiments with diabetic rats

All animal experiments were conducted in accordance with the guidelines of the Kyushu Institute of Technology and approved by the Animal Care and Use Committee of our institution.

Diabetes was induced in healthy rats (8 weeks old; Jcl:SD strain, male; CLEA Japan Inc., Tokyo, Japan), and BGC was measured as previously described [17]. Hair on the dorsal skin of diabetic rats induced by an intraperitoneal injection of STZ (65 mg/kg in ice-cold 20 mM sodium citrate buffer, pH: 4.6) was removed 48 h prior to the experiment. Insulin-loaded dBB MN (25% PVP) was applied to the shaved area using a spring-driven applicator and maintained for 10 s. Subsequently, the MN was fixed with surgical tape (Multipore™ Dry, 3 M, MN, USA) on the skin for 6 h. The BGC in a drop of blood collected from the tail vein was measured using a Medisafe^®^ Mini GR-102 (Terumo Co., Tokyo, Japan). After the experiments, the skin surface to which the dBB MN array had been applied was visually observed. To ascertain the efficacy of insulin-loaded dBB MN, BGCs of rats treated with 200 IU insulin solution (soaked in cotton) and treated with the MN array without insulin were used as controls.

## Results and discussion

### Fabrication of dBB MN arrays

PVP and HA were selected as the dMN materials in this study. PVP is a water-soluble, inert, non-toxic, pH-stabile, and biocompatible polymer that has been widely used as an excipient in various pharmaceutical applications [22]. HA has also been established as a material for dMN fabrication [5, 23]. A mixture of polymers was used to microcast the dMN to adjust the hydrogel’s characteristics [8, 24]. We further attempted to fabricate a dMN array using a hydrogel mixture with polymer contents of 25 w/w% (content ratio is a 1:3 = HA:PVP), 40 w/w% (1:4 = HA:PVP), and 38 w/w% (8:30 = PVA:PVP) [18]. Because it was difficult to adjust the viscosity of the hydrogel mixture within the optimal range, air bubbles remained in the needles, and some needles were not fabricated. Although it is possible that the optimal mixing ratio of base polymers could be discovered through further investigation, the dBB MN array was fabricated using only a base polymer (HA or PVP) in this study.

Table 1 summarizes the needle height of the dBB MN and their viscosity at various polymer concentrations. Needle height was maximal at 4% HA (954.2 ± 8.6 µm, Fig. 1b) and 25% PVP (982.7 ± 2.4 µm, Fig. 1c). The shape of original male mold was well reproduced using 25% PVP; however, plate-shaped needles of 4% HA were noticeably thinner than those of the original mold (Fig. 1a). Moreover, the influence of water content on the shape of the dMN was measured to evaluate the dBB MN array. In brief, when 4% HA and 25% PVP were heated at 85˚C for 6 h after storage in a desiccator; the resulting water contents were 6.25 ± 1.46% and 2.16 ± 1.42%, respectively. The MN height after drying decreased to 883.0 ± 8.2 µm for 4% HA and 936.3 ± 16.9 µm for 25% PVP, indicating that 4% HA dBB MNs would be affected by the drying that would occur during long-term storage.

**Table 1:** Needle height and viscosity in various concentrations of polymers

	Concentration [%]	Needle height [µm]	Viscosity [Pa∙s]
HA	2	932.2 ± 4.2	1.9
	4	954.2 ± 8.6	9.4
	6	932.8 ± 4.0	> 10.0
PVP	15	953.6 ± 2.8	3.4
	20	956.1 ± 9.6	5.4
	25	982.7 ± 2.4	8.5
	30	941.1 ± 3.9	> 10.0

The viscosities of 4% HA and 25% PVP were 9.4 Pa∙s and 8.5 Pa∙s, respectively. For the polymer concentrations with viscosities ≥ 10.0 Pa∙s (6% HA and 30% PVP) and ≤ 5.0 Pa∙s (2% HA and 15% PVP), the needle height was ≤ 950 µm. The needle tips did not have sharp edges when the hydrogel had a viscosity of > 10.0 Pa∙s. Hydrogels of viscosity > 10.0 Pa∙s do not enter into the micro-hole of the PDMS mold due to the high geometric complexity of the dBB MN, resulting in blunting of the needle tips. Conversely, the MNs shrunk when the water in the hydrogels of viscosity ≤ 5.0 Pa∙s evaporated. The property of the hydrogel needed to fabricate the dBB MN array was found to be in the range of 8–9 Pa∙s in viscosity. Therefore, the viscosity of the hydrogel influenced the needle height and sharpness of the dBB MN.

### Evaluation of dBB MN arrays

**Fig. 2:**
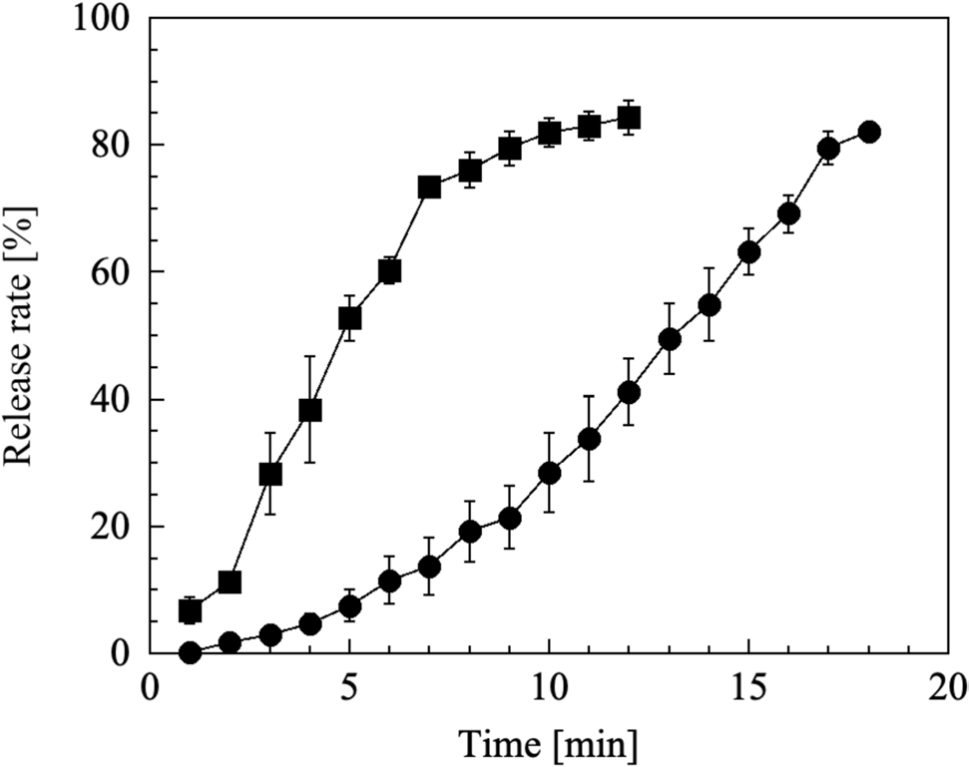
Release profiles of fluorescein from (■) 4% HA and (●) 25% PVP dBB MN array in PBS solution. The data points represent the mean ± standard deviation of three experiments

Figure 2 presents the release profiles of FL from 4% HA and 25% PVP dBB MN arrays in PBS. The dBB MN array with 4% HA completely dissolved within 7 min, whereas the 25% PVP dBB MN array exhibited a sustained release behavior and fully dissolved within 18 min. We assumed that the high polymer concentration in the hydrogel favored the maintenance of the shape and increased the hardness of the dBB MN.

**Fig. 3:**
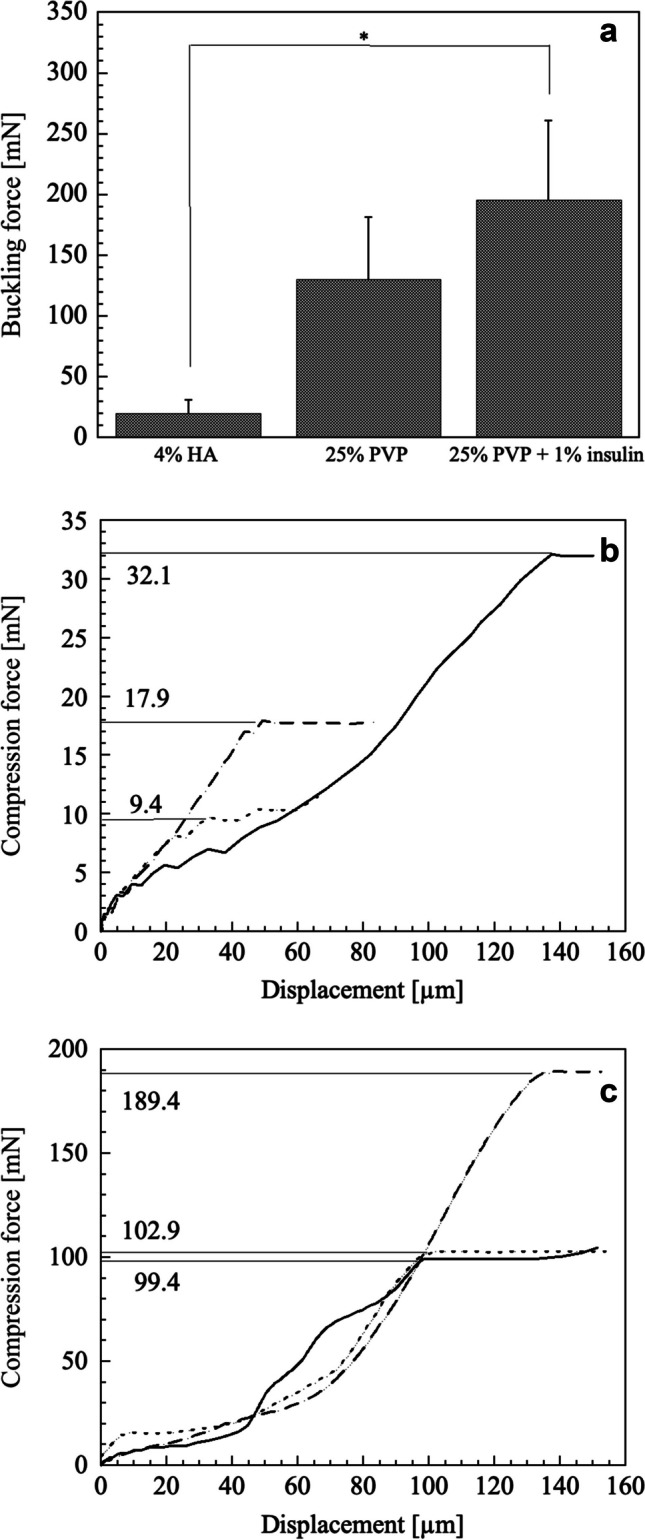
Hardness of dBB MN.** (a)** Comparison of buckling force for dBB MN. The compression curve at a single and different needles of (**b**) 4% HA and (**c**) 25% PVP. *; *p* < 0.05

We measured the needle hardness of 4% HA and 25% PVP dBB MN (Fig. 3a). The buckling force for 4% HA and 25% PVP was 19.8 ± 11.5 mN and 130.6 ± 51.0 mN, respectively (Fig. 3b and c). The buckling force of a single dMN mixed with two polymers was reported, and a hardness ranging from 100 to 480 mN of the dMN was sufficient to withstand the force required for skin puncture [11]. Thus, 4% HA may have no skin insertion capability and may therefore be unsuitable as a base polymer for dBB MNs.

**Fig. 4:**
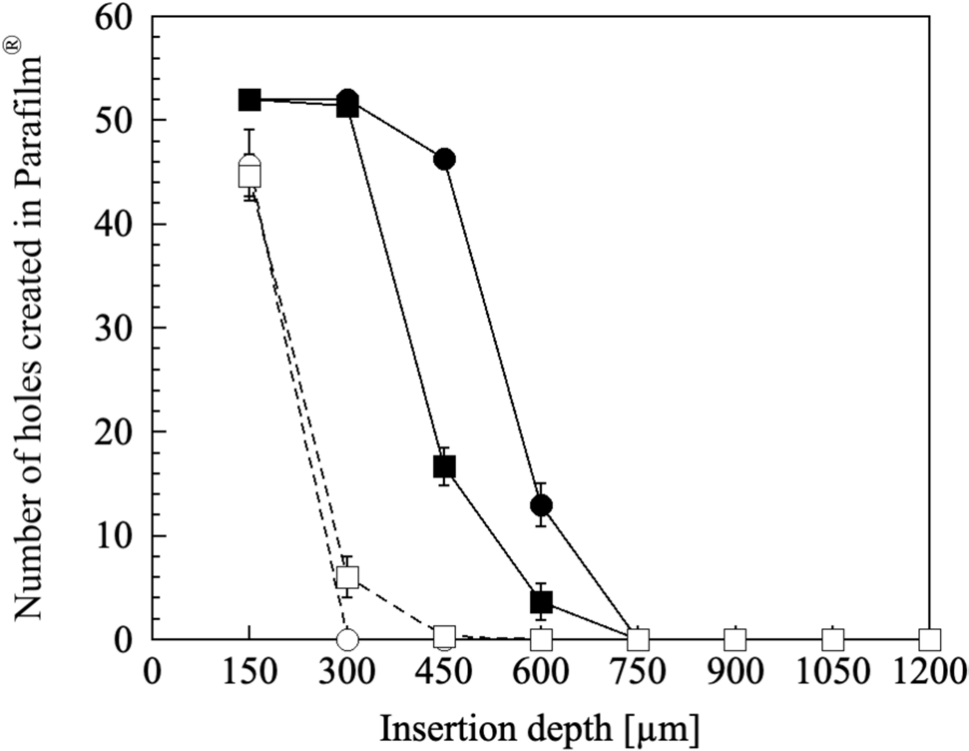
Insertion of dBB MN into eight layers of the artificial skin model, Parafilm^®^ M, using different application methods with a (circle) thumb and (square) spring-driven applicator. Solid (closed symbol) and dotted (open symbol) lines represent the 25% PVP and 4% HA, respectively. The data points indicate the mean ± standard deviation of three experiments

The insertion capability of the dBB MN was confirmed using an artificial skin model, Parafilm^®^ M, for which the thickness of one layer was 150 µm (Fig. 4). Application of the 25% PVP dBB MNs using either the thumb or an applicator resulted in all needles penetrating through the second layer (300 µm), which no needles reached the fifth layer (750 µm). With insertion force (17–19 N) of applicator, dBB MN formed micro-holes to penetrate the SC and the needle tips may reach the dermis. Conversely, some needles of the 4% HA were unable to puncture even the first layer (150 µm), and the insertion capability of 4% HA was clearly inferior compared to that of 25% PVP. Based on the results of fabrications and evaluation experiments, we decided to use 25% PVP as the material of dBB MN in the subsequent experiments.

### Skin insertion and transdermal delivery capability of dBB MN arrays

**Fig. 5:**
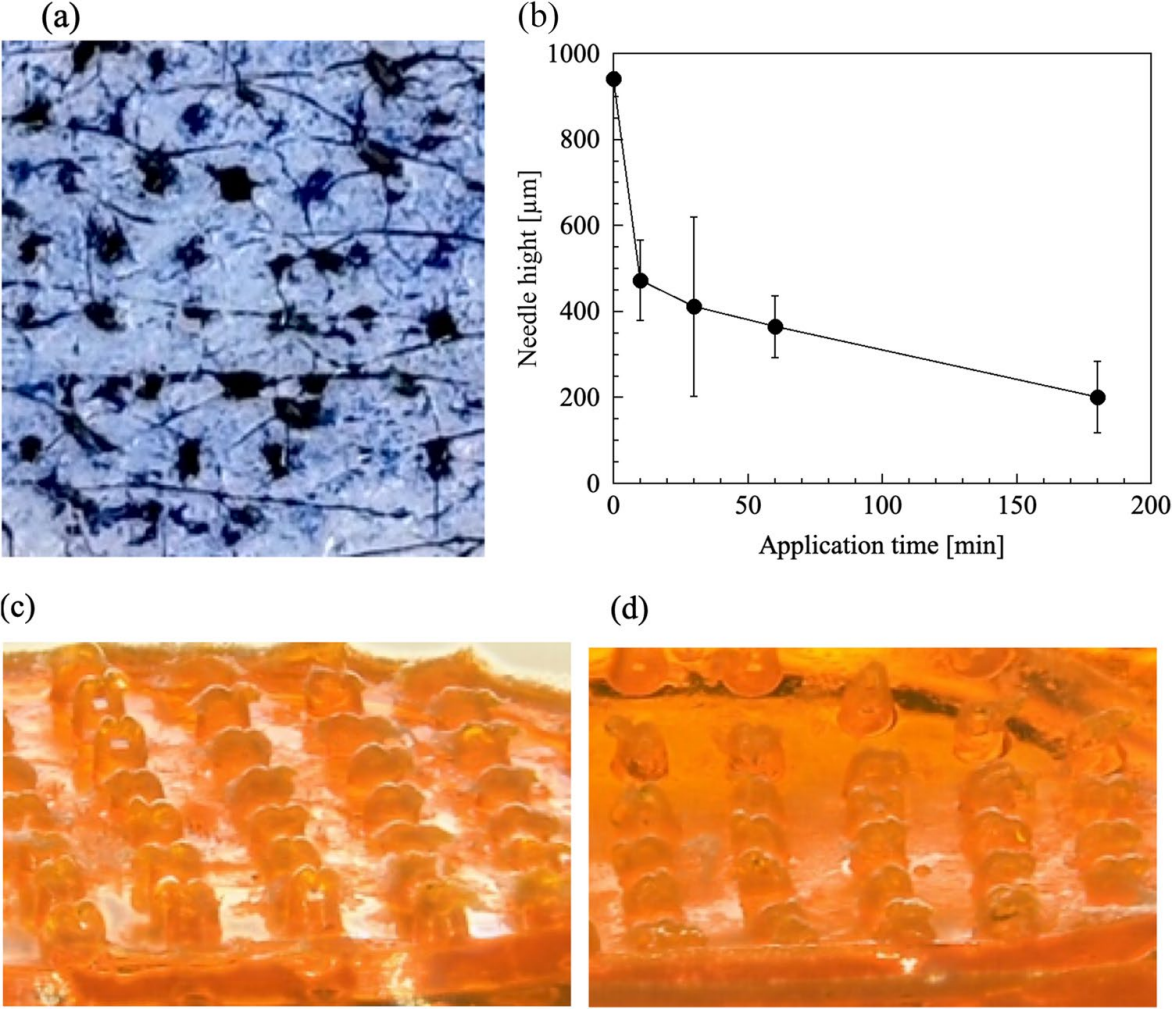
Skin insertion capability and dissolution experiment.** (a)** Photograph of swain skin surface treated 2 w/w% bromophenol blue after insertion of dBB MN. (**b**) Relationship of skin application time and needle height and the photographs of dBB MN array at (**c**) 10 and (**d**) 180 min after skin insertion

The skin insertion capability and dissolution experiment in a biological skin with interstitial fluid of dBB MN was assessed in vitro using swain skin. Figure 5a presents photographs of the skin surface treated 2 w/w% bromophenol blue solution to clarify the micro-holes after skin insertion. Micro-holes were created with the skin application of dBB MN array. Figure 5b presents the relationship between application time and needle height. The MN height decreased to 471.6 ± 54.1 µm within 10 min after insertion; subsequently, it decreased at a constant rate and was 200.4 ± 48.2 µm at 180 min. Two thin plate-shaped needles of dBB MN inserted in the SC would be dissolved within approximately 10 min (Fig. 5c), and then, the needle pedestal of the MN array was predicted to be dissolved slowly (Fig. 5d).

**Fig. 6:**
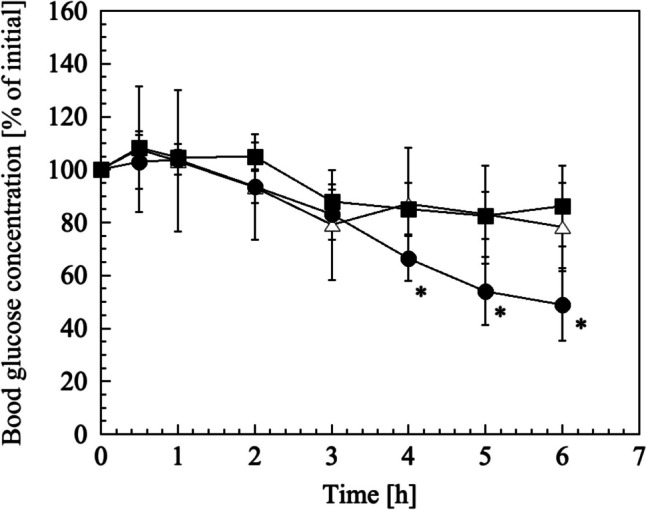
Time courses of blood glucose concentration in diabetic rats with (●) insulin-loaded dBB MN, (■) dBB MN without insulin, and (△) insulin solution on the intact skin. The data points indicate the mean ± standard deviation of six experiments. *; *p* < 0.05 compared to insulin solution at the same time-point

The buckling force of 25% PVP with insulin (195.6 ± 65.3 mN) was 1.50 times greater than that without insulin (Fig. 3a), which may result in insulin filling the space among PVP molecules. The time course of BGC in diabetic rats is shown in Fig. 6. No significant difference was observed between the BGCs of rats treated with the insulin solution and those treated with MN without insulin. In contrast, BGC decreased slowly and significantly after 3 h of application of the insulin-loaded dBB MN array (*p* < 0.05, two-tailed Student’s t-test with insulin solution). When the volume of the dBB MN array was calculated using 3D CAD software, the volume ratio of two needles and pedestal was estimated to be 0.31:0.69. To consider the water content (2.16%) of 25% PVP dBB MN array, the amount of insulin in all needles would need to be recalculated to 0.078 mg (2.0 IU). Thus, 0.024 mg (0.62 IU) of insulin was immediately delivered into the skin within 10 min of skin insertion, subsequently, a further 0.054 mg (1.38 IU) insulin in the pedestal, and large amounts of insulin in the base of the dMN array, were absorbed slowly into the skin over 3 h, resulting in a delayed reduction in BGC. No red spots or other skin abnormalities were observed on the dBB MN-applied site after experiments. The dBB MN array demonstrated sufficient ability to puncture rat skin and transdermally deliver a large-molecular-weight drug into the systemic circulation. However, it had the disadvantage of taking 3 h to reduce BGC. This issue could be addressed by optimizing the composition and concentration of biodegradable polymers.

## Conclusions

In this study, dBB MNs with pointed needle tips and sufficient hardness for skin insertion were fabricated using 25% PVP, with viscosity ranging from 8–9 Pa∙s, and the BGC in diabetic rats was significantly decreased by delivering a large-molecular-weight drug, insulin, into the systemic circulation. The two plate-shaped needles dissolved quickly in the SC after producing micro-holes, and the drug included in the needle pedestal and base of the dBB MN array penetrated the skin. However, the BGC did not show an immediately reduction after applying the dBB MN. This could be solved by investigating the quickly dissolution of biodegradable polymers in the skin and optimizing the needle geometry for deeper insertion into the skin. The dBB MN array offer a promising advancement in minimal invasive drug delivery systems, potentially improving patient compliance and access to therapies, particularly in underserved areas.

## Data Availability

The data and materials that support the findings of this study are available from the corresponding author, TH, upon reasonable request.
